# Evaluation of the Curative Effect of Umbilical Cord Mesenchymal Stem Cell Therapy for Knee Arthritis in Dogs Using Imaging Technology

**DOI:** 10.1155/2018/1983025

**Published:** 2018-05-15

**Authors:** Bei-ying Zhang, Bing-yun Wang, Shao-chuan Li, Dong-zhang Luo, Xiaoshu Zhan, Sheng-feng Chen, Zhi-sheng Chen, Can-ying Liu, Hui-qin Ji, Yin-shan Bai, Dong-sheng Li, Yang He

**Affiliations:** ^1^School of Life Science and Engineering, Foshan University, Foshan, Guangdong 528231, China; ^2^School of Veterinary Medicine, South China Agricultural University, Guangzhou 510642, China; ^3^Thai Yang Animal Hospital, Guangzhou 510000, China

## Abstract

**Objective:**

The aim of this study was to assess the efficacy of canine umbilical cord mesenchymal stem cells (UC-MSCs) on the treatment of knee osteoarthritis in dogs.

**Methods:**

Eight dogs were evenly assigned to two groups. The canine model of knee osteoarthritis was established by surgical manipulation of knee articular cartilage on these eight dogs. UC-MSCs were isolated from umbilical cord Wharton's jelly by 0.1% type collagenase I and identified by immunofluorescence staining and adipogenic and osteogenic differentiation *in vitro*. A suspension of allogeneic UC-MSCs (1 × 10^6^) and an equal amount of physiological saline was injected into the cavitas articularis in the treated and untreated control groups, respectively, on days 1 and 3 posttreatment. The structure of the canine knee joint was observed by magnetic resonance imaging (MRI), B-mode ultrasonography, and X-ray imaging at the 3rd, 7th, 14th, and 28th days after treatment. Concurrently, the levels of IL-6, IL-7, and TNF-*α* in the blood of the examined dogs were measured. Moreover, the recovery of cartilage and patella surface in the treated group and untreated group was compared using a scanning electron microscope (SEM) after a 35-day treatment.

**Results:**

Results revealed that the isolated cells were UC-MSCs, because they were positive for CD44 and negative for CD34 surface markers, and the cells were differentiated into adipocytes and osteoblasts. Imaging technology showed that as treatment time increased, the high signal in the MRI T2-weighted images decreased, the echo-free space in B ultrasonography images disappeared basically, and the continuous linear hypoechoic region at the trochlear sulcus thickened. On X-ray images, the serrate defect at the ventral cortex of the patella improved, and the low-density gap of the ventral patella and trochlear crest gradually increased in the treated group. On the contrary, the high signal in the MRI T2-weighted images and the echo-free space in B ultrasonography images still increased after a 14-day treatment in the untreated control group, and the linear hypoechoic region was discontinuous. On the X-ray images, there was no improvement in the serrate defect of the ventral cortex of the patella. Results for inflammatory factors showed that the blood levels of IL-6, IL-7, and TNF-*α* of the untreated control group were significantly higher than those of the treated group (*P* < 0.05) 7–14 days posttreatment. The result of SEM showed that the cartilage neogenesis in the treated group had visible neonatal tissue and more irregular arrangement of new tissue fibers than that of the untreated control group. Furthermore, more vacuoles but without collagen fibers were observed in the cartilage of the untreated control group, and the thickness of the neogenetic cartilage in the treated group (65.13 ± 5.29, 65.30 ± 5.83) and the untreated control group (34.27 ± 5.42) showed a significant difference (*P* < 0.01).

**Conclusion:**

Significantly higher improvement in cartilage neogenesis and recovery was observed in the treated group compared to the untreated control group. The joint fluid and the inflammatory response in the treated group decreased. Moreover, improved recovery in the neogenetic cartilage, damaged skin fascia, and muscle tissue around the joints was more significant in the treated group than in the untreated control group. In conclusion, canine UC-MSCs promote the repair of cartilage and patella injury in osteoarthritis, improve the healing of the surrounding tissues, and reduce the inflammatory response.

## 1. Introduction

Osteoarthritis (OA) is a common clinical disease in dogs affecting several tissues, including joint cartilage, subchondral bone, synovial membrane, and tendons [1]. OA is usually caused by injury, old age, and hereditary factors. Radiographic hallmarks of cartilage pathology include mainly joint space narrowing, subchondral sclerosis, subchondral cysts, osteophyte formation, and chronic inflammation of ligaments [2–5]. With the increasing occurrence of OA in dogs, research on canine OA has been increased in the past decade [6–12].

Among dogs over one year old, almost one out of every five has some degree of OA. Additionally, the morbidity in OA increases with age, and about 95% of OA cases occur in dogs over five years with dogs older than 10 years accounting for 50% of canine OA cases [13]. OA induces joint swelling, pain, deformation, and effusions, causing motor function obstacles and damage to health and welfare of the pet dogs [14].

Currently, the exact causes of OA and effective treatment remain elusive. Common treatments of OA have mainly focused on relieving pain and restoring joint function [15–18]. At present, many studies have shown that mesenchymal stem cells (MSCs) from different sources have significant effects on the regeneration and maintenance of cartilage in OA. Intra-articular injection of MSCs for cartilage restoration may become a new cell therapy for OA [19–21]. But the efficacy and mechanism of MSCs in the treatment of canine OA remain unclear [3]. Umbilical cord mesenchymal stem cells (UC-MSCs), which are isolated from the abandoned umbilical cord tissue of the neonate, have a strong ability to self-renew and the potential to differentiate into adipocytes, bone cells, nerve cells, hepatocytes, and myocytes [22, 23]. Moreover, UC-MSCs have many advantages, such as abundant resource, easy and noninvasive acquisition, low immunogenicity, strong proliferation ability, and no ethical controversy. Accordingly, UC-MSCs have become ideal seed cells for tissue restoration and organ reconstruction and are widely used in regenerative medicine [24–30].

Since OA is a ubiquitous problem, veterinarians need an accurate method to diagnose the disease precisely as early as possible. Imaging technologies, including X-ray, MRI, arthroscopy, and ultrasonography are widely used to check the joint lesions. X-ray is a routine examination of joint lesions which can assess the osteogenesis and the joint interspace; MRI offers a clear display of articular soft tissue lesions, with improved imaging quality.

Higher-quality images of the intra-articular synovial fluid, articular cartilage, and soft tissue around the joints are also observed on MRI/X-ray, while ultrasound has the advantage of displaying different degrees of the synovium thickening. In recent years, the application of high-frequency ultrasonography as a supplement to X-ray when evaluating complex joints (such as the knee) in joint disease has been gradually emphasized [13, 31, 32].

This study established a canine model of knee osteoarthritis by surgical manipulation and observed the effect of UC-MSC treatment on OA by X-ray, MRI, B-ultrasonography, and SEM, which could provide experimental evidence for the clinical treatment and imaging diagnosis of UC-MSCs in canine OA.

## 2. Materials and Methods

### 2.1. Animals

Eight healthy Chinese garden dogs, ranging from 5 to 6 months old and weighing from 7 to 8 kg, were obtained from Guangdong Medical Laboratory Animal Center. Animals were randomly divided into 2 groups: untreated control and treated groups (each group has 4 dogs, half of them males and half females). They were housed in individual cages and fed a standard diet with free access to drinking water. This study was approved by the Foshan University Veterinary Science Laboratory Animal Ethical Committee.

#### 2.2. Preparation of Canine UC-MSCs

Umbilical cord Wharton's jelly fragments were digested by 0.1% I type collagenase (Sigma, USA) [23, 33]. After filtration and centrifugation, canine UC-MSCs were cultured in OriCell mesenchymal stem cell growth medium (Cyagen Biosciences) supplemented with 10% fetal bovine serum, 1% penicillin/streptomycin, and 1% L-glutamine in a 5% CO_2_ incubator at 37°C. Adherent UC-MSCs were subsequently passaged, and the medium was aspirated to remove nonadherent cells.

### 2.3. Identification of Canine UC-MSCs

#### 2.3.1. Immunofluorescence

Cells were fixed in 4% paraformaldehyde in PBS at RT for 30 min and rinsed with wash buffer (PBS). Cells were then permeabilized with 0.1% Triton X-100 in PBS at RT for 30 min and rinsed with wash buffer (PBS). Blocking of nonspecific antibody interaction was carried out using a 10% fetal bovine serum solution. Blocking was carried out for 30 min. Primary antibodies of rabbit anti-CD34 antibody and mouse anti-CD44 antibody (Abcam) were added separately overnight at 4°C in the dark. After washing, cells were incubated for 30 min at RT with FITC-labeled secondary antibodies (FITC-labeled goat anti-rabbit IgG or FITC-labeled goat anti-mouse IgG). Cells were washed and counterstained with 1 *μ*g/mL DAPI prior to imaging.

#### 2.3.2. In Vitro Adipogenic and Osteogenic Differentiation

The third generation cells were seeded into the 24-well plates at a density of 10^4^ cells/mL and incubated in normal growth medium. When the cells reached 70% confluence, the growth medium was replaced with the DMEM adipogenic medium containing 10% fetal bovine serum, 1 *μ*mol/L dexamethasone, 10 *μ*g/mL insulin, and 200 *μ*mol/mL indomethacin. The induction medium was changed every 3 days in the induction group; meanwhile, the cells in the control group were continually fed with fresh growth medium. Cells were stained with Oil Red O for 30 min after the cells were induced for 3 weeks.

The third generation cells were seeded into the 24-well plates at a density of 10^4^ cells/mL and incubated for 24 h in normal growth medium. Then, the growth medium was replaced with DMEM osteogenic medium containing 10% fetal bovine serum, 0.1 *μ*mol/L dexamethasone, 10 mmol/L sodium glycerophosphate, and 50 *μ*mol/L ascorbic acid in the induction group, and cells in the control group were continually fed with the growth medium. Cells were identified by alizarin red staining two weeks after induction.

#### 2.4. Surgical Procedures of the OA Model

After induction with intravenous propofol (4 mg/kg), 8 dogs were anesthetized with 3% isoflurane in a mixture of oxygen and nitrous oxide delivered endotracheally. Surgery was carried out through a 2 to 3 cm medial incision close to the ligamentum patellae in the left knee. Special care was taken to prevent bleeding and soft tissue damage as much as possible. Before the surgery, the surgeon wore surgical sterile supplies; the surgical area was shaved, sterilized, laid with the sterile towel, and covered with a labeled surgical incision protective film.

In 8 dogs, the patella and cartilage of the left hind limb were damaged with a surgical drill after exposing the trochlea of the articular surface. Three transversal grooves were performed on the patella surface in contact with the weight-bearing parts of the femoral condyles. The cartilage on the weight-bearing parts of the femoral condyle wound was polished until the removal of crest and the sulcus of the trochlea [5, 34–36]. After surgery, synovium, fasciae, and skin were sutured by *2-0 PGS*. The contralateral unoperated knee served as a control.

#### 2.5. Postsurgical Treatment

The operated leg was not immobilized, and dogs were allowed to move freely in their cages. The injection schedule is illustrated in Table 1. The left hind limb was shaved prior to the surgical preparation of the skin. The syringe containing 1 × 10^6^ UC-MSCs/mL of normal saline was prepared sterilely and shaken gently prior to the injection [37, 38]. The syringe needle was pierced through the skin along the inferior patella until it reached the hypoechoic zone (the cavitas articularis containing joint fluid) of the left limb using B ultrasonography observation, then 1 mL of UC-MSC suspension was injected into the cavitas articularis of the treated group and 1 mL normal saline was injected into the cavitas articularis of the untreated control group.

The experimental animals received antibiotics (ampicillin 20 mg/kg during the first 4 days after surgery, cephalosporin 0.1 mL/kg during the 5th–7th days after surgery) for 7 days.

#### 2.6. Observation of the Left Hind Limb by Imaging Technology

On days 3, 7, 14, and 28 posttreatment, dogs were anesthetized for MRI, B ultrasonography, and X-ray evaluation with routine anesthetic protocols.

The MRI examinations were performed using a VET 0.3 T MRI unit (Ningbo Xingaoyi Magnetism Co. Ltd.) with small animal joint scanning sequence and knee coil. The situation of the joint effusion, inflammation, neonatal patella ventral, and cartilage pulley ridge and the peripheral structure of the knee was observed. MRI transverse relaxation (T2) of the articular cartilage reflects the water content, collagen content, and collagen fiber orientation in the ECM, with longer T2 values thought to represent cartilage degeneration [39–45]. The white area on the MRI T2-weighted images represented a high signal indicating an inflammatory response.

The B ultrasonography examinations were performed using a MyLab-20 unit (Baisheng, Italy). The dogs were laid in a supine position, and their left knees were scanned with 135-degree B ultrasonography to observe the situation of the joint effusion and the continuity of the articular cartilage. On B ultrasonography images, echo-free space represented an effusion and the continuous linear hypoechoic region at the trochlear sulcus represented a cartilage [46].

The X-ray examinations were performed using a 20 kW high-frequency X-ray machine (Shanxi Vanke) and a digital radiography (DR) system (Kangzhong, Hangzhou). The dogs were placed in a right lateral position, then their left hind limbs were focused and scanned by X-ray to observe the defects of the patella ventral and cartilage and the situation around the joints.

#### 2.7. Inflammatory Factor Measurements

On days 3, 7, 14, and 28 posttreatment, 5 mL blood of the examined dog was collected from the lateral saphenous vein of the hind limb or the medial cephalic vein of the forelimb to the anticoagulant tube and subsequently centrifuged at 2500 rpm/min for 10 min for plasma separation. Finally, the ELISA kits (Nanjing Jiancheng Bioengineering Research Institute) were used to detect IL-6, IL-7, and TNF-*α* in dogs.

#### 2.8. Observation of the Growth Status of the Patella and Cartilage by Scanning Electron Microscopy

On day 35 posttreatment, two dogs of the treated group and a dog of the untreated control group were sacrificed. Their excised patella cartilage was quickly immersed in an appropriate container filled with a primary fixative (2.5% glutaraldehyde (GA) in 0.1 M PBS buffer, pH 7.4) for a minimum of 1 h at room temperature. The specimens were fixed at 4°C for over 3 h, then dried, coated, and mounted for SEM analysis.

### 2.9. Statistical Analysis

SPSS17.0 software was used for statistical analysis. The data were expressed as mean ± standard deviation (M ± SD). Comparison between groups was analyzed by ANOVA. A *P* value of less than 0.05 was considered statistically significant.

## 3. Results

### 3.1. Immunofluorescence Assay

**Figure 1:**
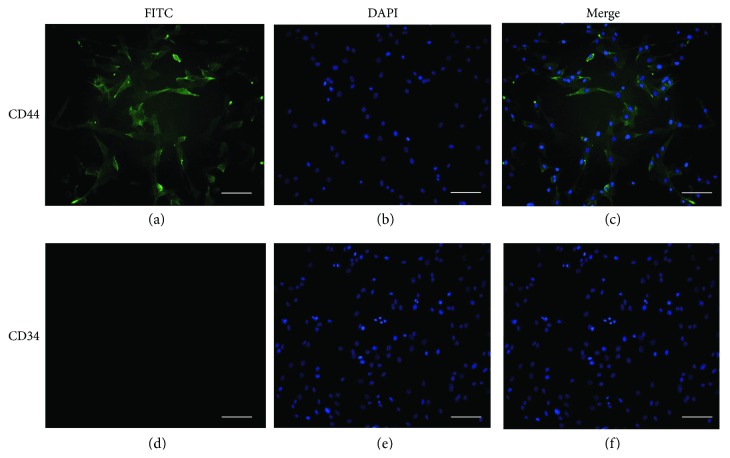
Immunofluorescence assay of UC-MSCs (scale bars = 100 *μ*m): (a) cells incubated with anti-CD44 antibody and FITC-labeled goat anti-mouse IgG; (b, e) counterstained by DAPI; (c) the merge image of (a) and (d), cells incubated with anti-CD34 antibody and FITC-labeled goat anti-rabbit IgG; (b, f) the merge image of (d) and (e).

Cells incubated with anti-CD44 antibody and FITC-labeled goat anti-mouse IgG showed a green fluorescence in the membrane (Figure 1(a)), and the nucleus was stained blue by DAPI (Figure 1(b)) indicating that the isolated cells were CD44 positive (Figure 1(c)). Similarly, the cells incubated with anti-CD34 antibody and FITC-labeled goat anti-rabbit IgG displayed no fluorescence in the cell membrane (Figure 1(d)); only the nucleus was stained blue by DAPI (Figure 1(e)), which showed that the expression of CD34 was negative in these cells (Figure 1(f)).

### 3.2. Adipogenic and Osteogenic Differentiation

**Figure 2:**
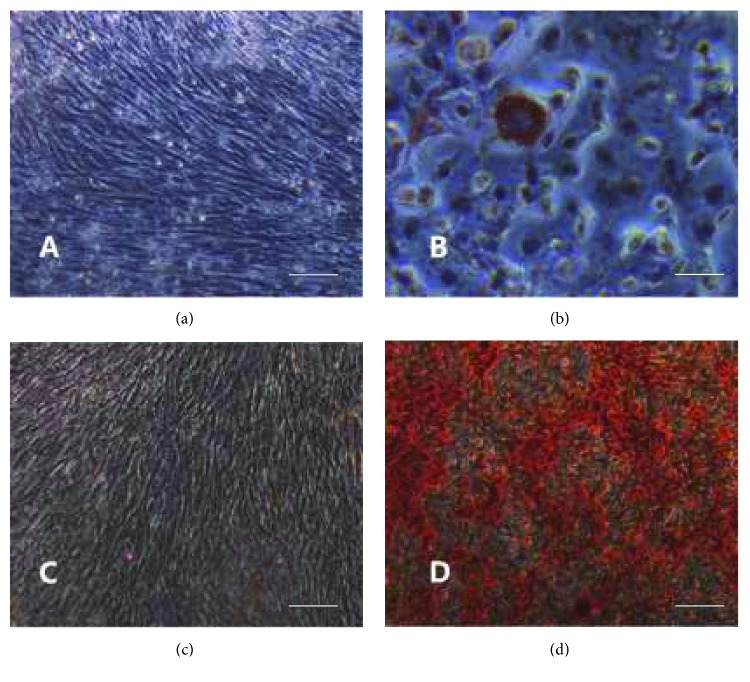
Adipogenic and osteogenic differentiation of UC-MSCs (scale bars = 100 *μ*m): (a) Oil Red O staining of UC-MSCs in the control group, (b) Oil Red O staining of UC-MSCs grown in adipogenic medium, (c) alizarin red staining of UC-MSCs in the control group, and (d) alizarin red staining of UC-MSCs grown in osteogenic medium.

Induction results showed that cells in both control groups grew in a long spindle shape and the confluent cells had obvious directionality in a swirly or reticular growth that could not be stained by Oil Red O and alizarin red (Figures 2(a) and 2(c)). In the induction groups, lipid droplets were observed in the cytoplasm and stained red by Oil Red O staining after adipogenic differentiation (Figure 2(b)). Calcium nodules were observed and stained red by alizarin red in the induction groups after osteogenic differentiation (Figure 2(d)).

### 3.3. MRI Assessment

**Figure 3:**
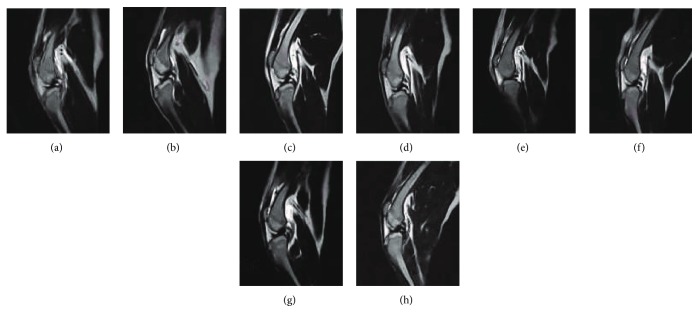
Magnetic resonance imaging of canine arthritis after the treatment: (a) 3 days after cell therapy, (b) 7 days after cell therapy, (c) 14 days after cell therapy, (d) 28 days after cell therapy, (e) 3 days after treatment in the untreated control group, (f) 7 days after treatment in the untreated control group, (g) 14 days after treatment in the untreated control group, and (h) 28 days after treatment in the untreated control group.

MRI T2-weighted images showed that the tissues around the canine joints were disorganized, and there was a high-signal area between the distal femur and subcutaneous area in both treated and untreated control groups at 3 days posttreatment (Figures 3(a) and 3(e)). The high-signal areas of the distal femoral and subcutaneous peripheral joint in the treated group were gradually weakened and the signal of joint fluid and cartilage between the patella and trochlear crest appeared at 7–14 days posttreatment (Figures 3(b) and 3(c)). Moreover, the high-signal areas of the distal femoral and subcutaneous peripheral joint disappeared in the treated group at 28 days posttreatment. The patella ventral became smooth and there was a signal between the patella and the trochlear crest (Figure 3(d)). But in the untreated control group, the high-signal areas between the distal femoral and subcutaneous peripheral joint did not weaken at 28 days posttreatment (Figures 3(f)–3(h)). Meanwhile, the signal of the patella ventral remained disorganized at 7–14 days posttreatment (Figures 3(f) and 3(g)) and there was no marked improvement in the patella ventral and the cartilage trochlear crest (Figure 3(h)).

### 3.4. B Ultrasonography Assessment

**Figure 4:**
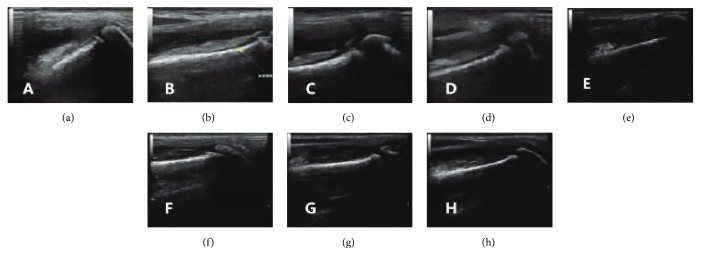
B ultrasonography assessment of canine arthritis after the treatment: (a) 3 days after cell therapy, (b) 7 days after cell therapy, (c) 14 days after cell therapy, (d) 28 days after cell therapy, (e) 3 days after treatment in the untreated control group, (f) 7 days after treatment in the untreated control group, (g) 14 days after treatment in the untreated control group, and (h) 28 days after treatment in the untreated control group.

B ultrasonography images showed that there was an echo-free space in the cavitas articularis and a discontinuous linear hypoechoic region at the trochlear sulcus at 3 days posttreatment. Additionally, the joint tissues had the existence of medium- and low-clutter echoes, and the echo levels of the skin fascia and muscle tissue were not clear in the two groups (Figures 4(a) and 4(e)). In the treated group, the intra-articular echo-free space was reduced, and the linear hypoechoic region of the trochlea was visible and continuous at 7 days posttreatment (Figure 4(b)). The echo-free space in the cavitas articularis disappeared, and the linear hypoechoic area was continuous and thickened at 14 days posttreatment (Figure 4(c)). The hypoechoic region of the trochlea was visible, and the linear hypoechoic area was continuous, with increased thickness. The medium- and low-clutter echoes around the joint disappeared, and the skin fascia and muscle tissue echo layer were clearer at 28 days posttreatment (Figure 4(d)). Meanwhile, the intra-articular echo-free space of the untreated control group still existed, and there was no linear hypoechoic region at the trochlea sulcus at 7, 14, and 28 days posttreatment (Figures 4(f)–4(h)).

### 3.5. X-Ray Assessment

**Figure 5:**
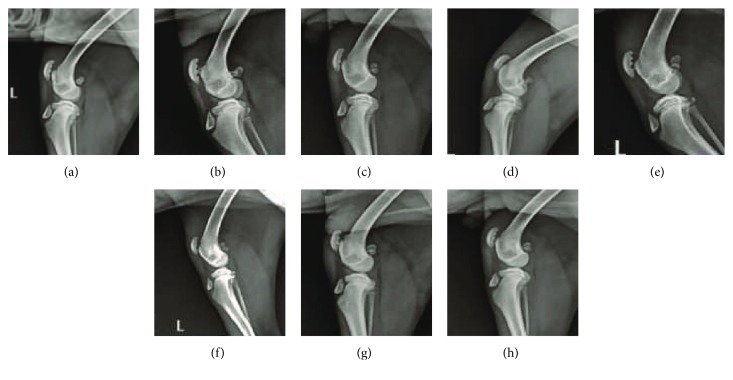
X-ray scan images of canine arthritis after treatment: (a) 3 days after cell therapy, (b) 7 days after cell therapy, (c) 14 days after cell therapy, (d) 28 days after cell therapy, (e) 3 days after treatment in the untreated control group, (f) 7 days after treatment in the untreated control group, (g) 14 days after treatment in the untreated control group, and (h) 28 days after treatment in the untreated control group.

X-ray images showed that there were three grooves on the patella ventral of the left knee, and the low-density gap between the patella ventral and pulley ridge was not found in both the treated (Figures 5(a) and 5(b)) and untreated control groups (Figures 5(e) and 5(f)) at 3 and 7 days posttreatment. After 14 days posttreatment, the grooves of the patella ventral of the left hind limb were improved in the treated group, and the low-density gap between the patella ventral and pulley ridge was enlarged (Figure 5(c)). The cortical bone defects of the patella ventral on the left knee were continually improved, and the trochlea was smoother in the treated group at 28 days posttreatment. In comparison between the two groups, the low-density gap between the patella ventral and trochlea was increased at 14 days posttreatment (Figure 5(d)).

In the untreated control group, extreme defects of the patella ventral were noticed at 14 days posttreatment (Figure 5(g)). After 28 days of treatment, the grooves in the untreated control group remained observed. Moreover, there was no improvement in the low-density gap between the patella ventral and trochlea (Figure 5(h)).

### 3.6. Inflammatory Factors

**Figure 6:**
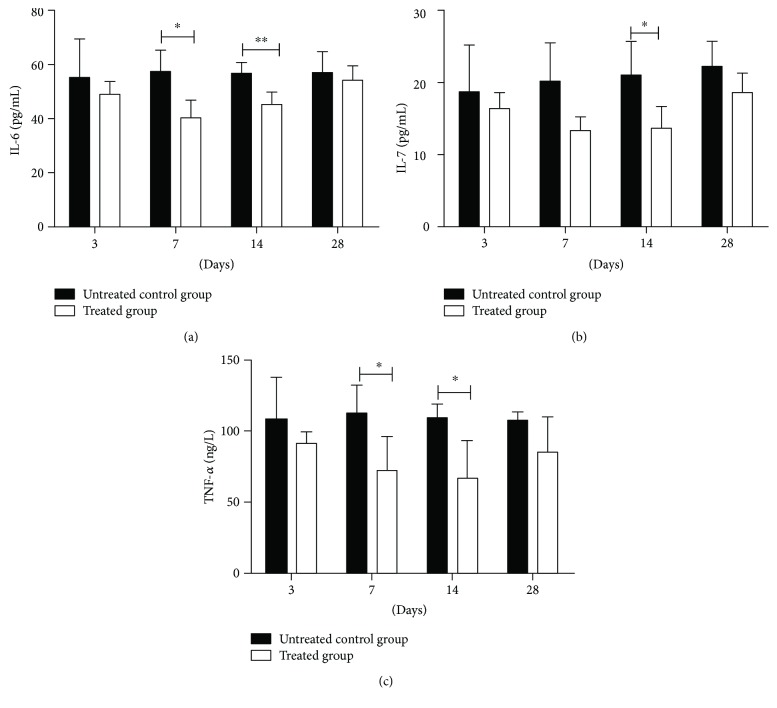
The blood levels of IL-6, IL-7, and TNF-*α* in dogs (mean ± SD, *n* = 4): (a) the blood level of IL-6 in dogs on days 3, 7, 14, and 28 posttreatment; (b) the blood level of IL-7 in dogs on days 3, 7, 14, and 28 posttreatment; and (c) the blood level of TNF-*α* in dogs on days 3, 7, 14, and 28 posttreatment. ^∗^*P* < 0.05 and ^∗∗^*P* < 0.01.

The ELASE results showed that there was no significant difference in the levels of IL-6, IL-7, and TNF-*α* between the untreated control group and the treated group on the 3rd and 28th days after the treatment. However, the levels of IL-6 and TNF-*α* in the untreated control group were significantly higher than those in the treated group after 7 days posttreatment (*P* < 0.05). Specifically, 14 days posttreatment, the levels of IL-6 had a very significant difference in the two groups (*P* < 0.01), and levels of IL-7 and TNF-*α* both had a significant difference between the untreated control group and the treated group (*P* < 0.05) (Figures 6(a)–6(c)).

### 3.7. SEM Assessment

**Figure 7:**
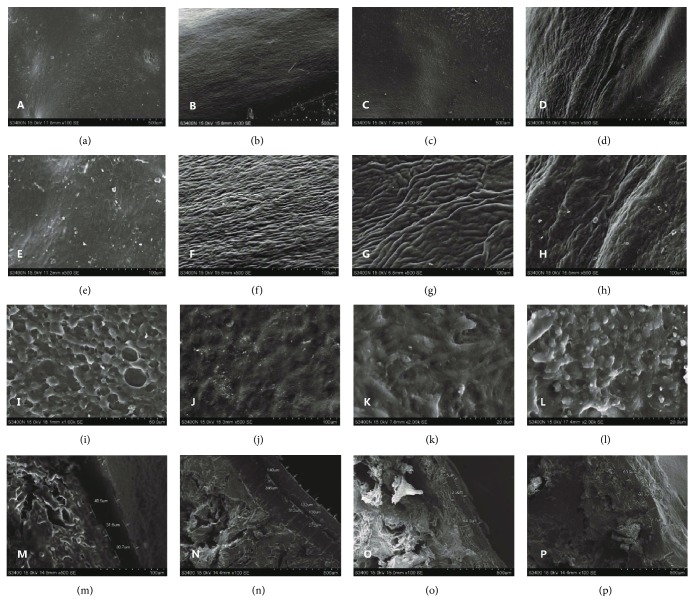
The canine knee cartilage images by SEM: (a) cross section of the untreated control group (×100 SE), (b) cross section of the normal control group (×100 SE), (c) cross section of the central neogenetic cartilage in the treated group (×100 SE), (d) cross section of the surrounding neogenetic cartilage in the treated group (×100 SE), (e) cross section of the untreated control group (×500 SE), (f) cross section of the normal control group (×500 SE), (g) cross section of the central neogenetic cartilage in the treated group (×500 SE), (h) cross section of the surrounding neogenetic cartilage in the treated group (×500 SE), (i) cross section of the untreated control group (×1000 SE), (j) cross section of the normal control group (×500 SE), (k) cross section of the central neogenetic cartilage in the treated group (×2000 SE), (l) cross section of the surrounding neogenetic cartilage in the treated group (×2000 SE), (m) vertical section of the untreated control group (×100 SE), (n) vertical section of the normal control group (×100 SE), (o) vertical section of the treated group *a* (×100 SE), and (p) vertical section of the treated group *b* (×100 SE).

SEM images showed that the surface of the cartilage in the normal control group was smooth (Figure 7(b)). The cartilage was composed of many collagen fiber bundles with a dense vertical arrangement of different thicknesses (Figure 7(f)) and the visible single or group cartilage lacuna (Figure 7(j)). In the untreated control group, the cartilage surface showed a vacuole-shaped damage (Figures 7(a) and 7(i)) and had no visible collagen fibers (Figure 7). In the treated group, there were some small protuberances on the central surface of the cartilage (Figure 7(c)). The neonatal cartilage, which was composed of many collagen fiber bundles in an irregular arrangement (Figures 7(g)–7(h)), grew from the surroundings to the central surface (Figure 7(d)). The neonatal tissue was disorganized (Figure 7(k)) and had the form of small protuberances (Figure 7(l)).

The thickness of the new cartilage tissue was measured by SEM (Figures 7(m)–7(p)). The data from Table 2 showed that the thickness of the new cartilage in the treated group was significantly higher than that in the untreated control group (*P* < 0.01).

**Figure 8:**
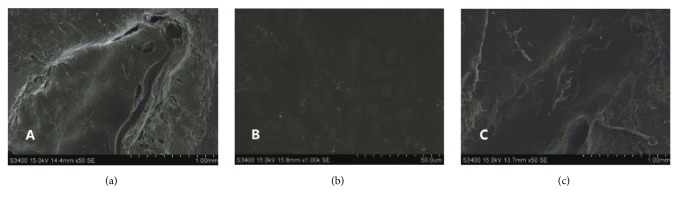
Observation of canine knee patella by SEM: (a) untreated control group (×50 SE), (b) normal control group (×1000 SE), and (c) treated group (×50 SE).

SEM images showed that the patella surface of the normal control group was flat, with no cracks (Figure 8(b)). There were neogenetic tissues at the defective patella in the untreated control and treated groups, but the defect of the untreated control group was more severe than that of the treated one (Figure 8(a)), while the healing parts in the treated group were smoother than those in the untreated control group (Figure 8(c)).

## 4. Discussion

MSC was first discovered from the bone marrow stroma by Pacini and Petrini. The proliferative adherent fibroblast colonies, which could differentiate into fibroblasts, chondrocytes, and osteoblasts *in vitro* and *in vivo*, were isolated from the cell suspensions of cultured human bone marrow (hBM) [47]. Recent studies have found that MSCs isolated and obtained from bone marrow, umbilical cord blood, fat, placenta, and umbilical cord were a colony of multiple endogenous progenitor cells from the mesoderm with the ability of self-renewal and multipotential differentiation into bone, fat, cartilage, and the musculoskeletal system and have a homing ability of migration to injured locations of the body [48, 49]. According to previous studies, MSCs express surface markers, such as CD13, CD44, CD73, CD90, CD105, and CD29, but they do not express the surface markers of other hematopoietic cell lines, for example, CD45, CD14, and CD34 [50–52]. Therefore, CD34 and CD44 were selected to identify the isolated cells by immunofluorescence staining.

MSCs play a role in immune regulation mainly by its paracrine actions in inflammatory response [17]. At present, many studies on MSC therapy have been carried out to treat osteoarthritis tendon and spinal cord injury, kidney and liver disease, and many other diseases in pets [53]. MSCs have been found to be the best regenerative cell therapy for degenerative musculoskeletal disorders such as OA [54]. In this study, the isolated cells from the umbilical cord Wharton's jelly were identified by immunofluorescence staining and adipogenic and osteogenic differentiation. The immunofluorescence result showed that the cells were positive for CD44 expression and negative for CD34 expression. Besides, these cells were successfully differentiated into adipocytes and osteoblasts. Results showed that the isolated cells typically had MSC characteristics.

Many studies have used MSCs to treat spontaneous OA models and artificially induced OA models (in small animals, including mice, rats, rabbits, and guinea pigs, and in large animals, e.g., dogs, goats, sheep, pigs, and horses). Results from these studies have shown that MSCs from bone marrow, fat, and cord blood can be effectively used to treat OA [31]. Lavigne et al. [55] have previously demonstrated the regenerative effect of BM-MSCs. Their results showed that the medial meniscus regeneration in the treated group was more significant compared to the untreated control group which had been injected with HA only. When BM-MSCs were used to treat a patient with meniscus and cartilage damage 24 weeks after the treatment, MRI observation showed that there was a significant increase in the volume of cartilage and meniscus, the range of motion increased, and the pain score decreased (Sampson et al. [54]).

Vilar et al. showed AD-MSC treatments (from fat) through intra-articular injection to treat 8 dogs with severe OA [56]. To study the efficacy of AD-MSCs, a force platform was used to measure the limb function. The results showed that the PVF and VI of the treated dogs significantly increased. Thus, AD-MSC treatment could reduce the claudication induced by OA. Furthermore, it was shown that the use of adjuvants, such as PRGF-Endoret could prolong the therapeutic effect of MSCs [56]. Although MSC treatment of animals with OA has a certain effect, there are still many unanswered questions concerning the effect and mechanism of MSC therapy in dogs with OA due to the limitations of the means and methods to detect the curative effect.

In this study, UC-MSCs were injected into the articular cavity using a canine OA model to treat canine OA, and the therapeutic effect and mechanism of MSCs were observed by MRI, B ultrasonography, X-ray, and electron microscopy. The results showed that joint effusion and the inflammatory reaction decreased 1 week after MSC therapy. Application of MSC therapy promoted the repair of cartilage and patella in OA-affected dogs and improved the healing of the surrounding tissue, and the effect of the MSCs was shown to last for 1 month. Lamo-Espinosa et al. [57] randomly divided 30 patients into a control group which was treated with HA alone, a low-dose group which received a combination of HA with 10 × 10^6^ autologous BM-MSCs, and a high-dose group to which both HA and a suspension of 100 × 10^6^ autologous BM-MSCs were administered. VAS and WOMAC were used to assess pain and function. The range of knee movement was measured in 12 months, and X-ray and MRI were used to observe joint damage. The results showed that VAS and WOMAC of the BM-MSC-injected groups were superior to the untreated control group, and there was a significant difference between the high-dose group and the untreated control group in 12 months (*P* < 0.01). X-ray examination showed that the width of the knee joint decreased compared to the high-dose group. MRI (WORMS) results showed that a slight reduction in joint damage was only found in the BM-MSC high-dose group, indicating that BM-MSCs could contribute to the improvement of OA clinically and functionally [57].

In this study, the experimental dogs were put to death to observe the cartilage improvement after 35 days of treatment. The growth of cartilage from the surroundings to the center was observed in both groups of the canine OA model, but the recovery rate of the treated group was substantially significant in comparison with the untreated control one. The neonatal part around the cartilage in the untreated control group was relatively thin—the bone beneath could still be seen—and the central formation of new tissue had small protrusions. While the neonatal cartilage layer was thicker in the treatment group, there was a very significant difference between the treatment group and the model group in the thickness of neonatal cartilage (*P* < 0.01).

OA may lead to progressive cartilage degeneration, which is characterized by softening, fibrillation, and erosion of the articular surface [3, 58, 59]. Scanning electron microscopy showed that the surface of the neonatal cartilage tissue was synaptic collagen fibers, which was different from the cartilage in the normal group. Vacuoles could be seen in the untreated control group with no collagen fibers, but irregular arrangement of new tissue fibers could be seen in the treated group.

The results indicated that UC-MSCs could promote the growth of chondrocytes effectively and relieve the OA-induced progressive cartilage degeneration. Furthermore, UC-MSCs could reduce the incidence of joint effusion and inflammation, increase the regeneration of cartilage tissue, and promote the recovery of the patella defects.

The metabolic activity of chondrocytes can be affected by many factors, the most important of which is proinflammatory cytokines and growth factors. It is known that inflammatory cytokines such as IL-1*α*, TNF-*α*, and IL-6 are upregulated during OA progression [3, 19, 60]. In this study, the results showed that inflammatory cytokines, such as TNF-*α*, IL-6, and IL-7 in the untreated control group were significantly higher compared to the treated group (*P* < 0.05).

These results showed that UC-MSCs could reduce the inflammatory response in OA by reducing the secretion of inflammatory factors and influence the proliferation and metabolic activity of chondrocytes. Recent studies have shown that there is a MSC niche in cartilage, indicating that AC chondrocytes can be derived from MSCs [3]. Previous studies have found that OA tissues in fibrous and nonfibrous regions exhibit cell aggregation effects, cell proliferation, and the aggregation in injured areas [61, 62]. Several studies have shown that the main benefit of MSCs is due to its paracrine activity [63]. The main function of chondrocytes in the superficial and central regions is to synthesize the extracellular matrices and proteoglycans consisting of types II, IX, and XI collagens [3]. MSCs can alter the cell signaling environment, increase the production of collagen type II, promote the migration of chondrocytes to the injured area, and repair the damage by synthesizing the lost extracellular matrix [64, 65]. Thus, MSCs may treat OA by adjusting the repair response of the joint rather than replacing the damaged area directly.

Based on the present trial and the results of this study, the possible mechanism of MSCs in the treatment of OA is by inhibiting the inflammatory response, reducing synovial fluid, and promoting the synthesis of extracellular matrix and chondrocyte proliferation and migration. Further research is needed to explore other mechanisms of the MSC therapy in OA.

## 5. Conclusion

Canine UC-MSCs promote the repair of cartilage and patella injury by osteoarthritis, improve the surrounding injury tissue of the joints, and reduce the inflammation response.

## Figures and Tables

**Table 1:** Injection schedules.

Group	Injections	Injection day (days after the surgical)						
		1	2	3	4	5	6	7
Untreated control group		Normal saline 1 mL	—	Normal saline 1 mL	—	—	—	—
	Antibiotics	Ampicillin 20 mg/kg			Cephalosporin 0.1 mL/kg			

Treated group	Cells	UC-MSC suspension 1 mL (1 × 10^6^cells/mL)	—	UC-MSC suspension 1 mL (1 × 10^6^cells/mL)	—	—	—	—
	Antibiotics	Ampicillin 20 mg/kg			Cephalosporin 0.1 mL/kg			

**Table 2:** The thickness of newborn cartilage in the OA model (mean ± SD, *n* = 3).

Group	Newborn cartilage thickness (*μ*m)	*P* values
Untreated control group	34.27 ± 5.42	
Treated group *a*	65.13 ± 5.29^∗∗^	*P* < 0.01
Treated group *b*	65.30 ± 5.83^∗∗^	*P* < 0.01

^∗∗^There is a very significant difference between two groups.
